# Environmental stress - what can we learn from chlorophyll *a* fluorescence analysis in woody plants? A review

**DOI:** 10.3389/fpls.2022.1048582

**Published:** 2022-12-14

**Authors:** Tatiana Swoczyna, Hazem M. Kalaji, Filippo Bussotti, Jacek Mojski, Martina Pollastrini

**Affiliations:** ^1^ Department of Environment Protection and Dendrology, Institute of Horticultural Sciences, Warsaw University of Life Sciences SGGW, Warsaw, Poland; ^2^ Department of Plant Physiology, Institute of Biology, Warsaw University of Life Sciences SGGW, Warsaw, Poland; ^3^ Department of Agriculture, Food, Environment and Forestry, University of Florence, Florence, Italy; ^4^ Twój Swiat Jacek Mojski, Łukow, Poland; ^5^ Fundacja Zielona Infrastruktura, Łukow, Poland

**Keywords:** forests, JIP-test, PAM fluorescence, shrubs, trees, urban trees

## Abstract

Chlorophyll *a* fluorescence (ChF) signal analysis has become a widely used and rapid, non-invasive technique to study the photosynthetic process under stress conditions. It monitors plant responses to various environmental factors affecting plants under experimental and field conditions. Thus, it enables extensive research in ecology and benefits forestry, agriculture, horticulture, and arboriculture. Woody plants, especially trees, as organisms with a considerable life span, have a different life strategy than herbaceous plants and show more complex responses to stress. The range of changes in photosynthetic efficiency of trees depends on their age, ontogeny, species-specific characteristics, and acclimation ability. This review compiles the results of the most commonly used ChF techniques at the foliar scale. We describe the results of experimental studies to identify stress factors that affect photosynthetic efficiency and analyse the experience of assessing tree vigour in natural and human-modified environments. We discuss both the circumstances under which ChF can be successfully used to assess woody plant health and the ChF parameters that can be useful in field research. Finally, we summarise the advantages and limitations of the ChF method in research on trees, shrubs, and woody vines.

## Introduction

Long-lived woody plants, i.e., trees and shrubs, build up their structure over the years and adapt it to environmental and climatic conditions; moreover, temporal variations in the length and intensity of periods of cold, heat, drought, etc., provide some flexibility in responding to environmental stressors (Kozlowski et al., 2012). From leaf emergence, woody plants tend to extend leaf life to the end of the season (deciduous species) or beyond (evergreen species), whereas, in herbaceous plants, leaf life is usually shortened due to shading of primary leaves and investment in newly emerging leaves (Diemer et al., 1992; Kikuzawa, 1995; Kikuzawa and Ackerly, 1999). In the early stages, seedlings and young trees (saplings) differ from mature specimens in terms of leaf structure and photosynthetic activity (Bond, 2000; Niinemets, 2002; Mediavilla et al., 2014). Because of their longevity, woody plants have a greater potential to recover from damage (Haukioja and Koricheva, 2000). The continuous (annual) growth of trees is under the control of growth regulators and biochemical and physical balances that tend to keep various processes and structures in equilibrium (Kozlowski et al., 2012).

In the face of environmental stress, woody plants have evolved various mechanisms to protect themselves from damage and adverse conditions (Bussotti and Pollastrini, 2021). These mechanisms operate on a plant-wide level. The results of experiments conducted under controlled conditions provide the basis for interpreting plant responses observed in the environment. Usually, a stressor is applied at a high intensity so that the stress response becomes evident and clear conclusions can be drawn (Kalaji et al., 2018). Most of these experiments are conducted on seedlings or small saplings, i.e., in the early stages of life. However, the complexity of factors affecting woody plants and the variability in the intensity of these factors during their life (extreme summers or winters, human-induced changes in the soil environment, etc.) can sometimes make it difficult to explain the background of the responses of the trees/shrubs studied (Swoczyna and Latocha, 2020). The complexity of environmental conditions may affect the magnitude and duration of the response to stress, e.g., limited access to nitrogen in the soil may increase the effect of drought stress, triggering a change in growth strategy and physiology (Ögren, 1988). Indeed, one type of response identified as a stress response, e.g., defoliation, may not be mirrored by another, e.g., reduced photosynthetic efficiency of the remaining leaves or shoots (Desotgiu et al, 2012b; Suchocka et al., 2021).

In recent decades, the diversity of chlorophyll *a* fluorescence (ChF) research has increased considerably, and for the last decade there has been a tremendous development in this discipline (Bąba et al., 2019). During this time, methods and protocols have been developed and tested, as well as instruments whose design and operating principles have been refined (Strasser et al., 2004; Stirbet and Govindjee, 2011; Tsimilli-Michael, 2020). This optical method, in contrast to, for example, time-consuming infrared gas exchange measurements or chemical analyses of collected samples, enables numerous non-destructive and non-invasive experiments on plants in which their photosynthetic properties are recorded in response to environmental conditions (Kalaji et al., 2014b). The available instruments are portable and can be used in field conditions, allowing the study of plants both in plantations and in natural or urban environments (Christen et al., 2007; Fini et al., 2009; Ugolini et al, 2012; Pollastrini et al., 2016b). In addition, the ChF method examines the efficiency of the photosynthetic apparatus, i.e., the current state or conformation of photosystems and their compounds, rather than the process of photosynthesis itself, which is why it is possible to perform measurements on detached leaves (Percival and Fraser, 2002). Advances in the development of easy-to-use equipment have expanded the application of the ChF technique in numerous research studies in agriculture, horticulture, arboriculture, forestry, and environmental studies, as well as in practical applications in commerce. The different techniques for measuring ChF provide specific parameters whose importance overlaps to some extent. Some review articles have already provided an overview of the application of ChF measurements in stress detection using different techniques: pulse amplitude modulated ChF (Baker and Rosenqvist, 2004; Murchie and Lawson, 2013), chlorophyll fluorescence imaging techniques (Baker and Rosenqvist, 2004; Baker, 2008; Gorbe and Calatayud, 2012), chlorophyll fluorescence induction curve analysis (OJIP analysis) based mainly on crop research (Kalaji et al., 2016) or forest research (Pollastrini et al., 2016b; Bussotti et al., 2020).

The measured ChF signal is mainly from PSII and is the re-emitted excess energy that was neither involved in photochemical processes nor dissipated as heat. Photochemistry, heat dissipation, and fluorescence are competing processes, so fluorescence measurements can be used to evaluate the balance between photochemistry and non-photochemical dissipation of absorbed light (Maxwell, 2000). Chlorophyll fluorescence measurements made directly on leaf samples provide numerous parametric data that allow deeper analysis of physiological processes associated with the light phase of photosynthesis. Fluorimeters with different operating principles are used for this purpose (Baker and Rosenqvist, 2004; Kalaji et al., 2014b; Banks, 2017; Padhi et al., 2021). Signals of chlorophyll fluorescence can be detected from samples previously adapted to darkness (when all photochemical reactions have been quenched) as well as from samples in ambient light. Separate protocols had to be developed for these two approaches. Adaptation of a leaf sample to darkness allows suppression of all light-dependent processes. For rapid exposure to actinic saturating light, two of the most commonly used values, F_0_ and F_M_, are determined and used to calculate the maximum efficiency of the photosystem II, F_V_/F_M_. The latter ratio has long been attractive for determining differences in photosynthetic performance between plants (Ögren, 1990; Percival, 2002).

Pulse amplitude modulated fluorimeters use actinic light (blue or red), which stimulates photosynthesis, and additional emitted measurement light, which is used to study the state of the photosynthetic system (Baker and Rosenqvist, 2004; Kalaji et al., 2014b). The measuring light is applied with constant pulse amplitude. The on and off switching of the actinic light is synchronised to be in the middle of the dark periods between the measurement light pulses and is used to evaluate the maximum fluorescence yield. Any non-modulated fluorescence signal (e.g., from daylight) is completely suppressed by the amplifier system in the PAM fluorimeter (Schreiber, 2004). PAM method allows evaluation of the so-called “photochemical quenching”, q_P_, which is related to photochemical energy utilisation by charge separation at the reaction centres of PSII. “Non-photochemical quenching”, a non-radiative dissipation of energy into heat, can be expressed in two ways, q_N_ (Schreiber, 2004) or NPQ (Bilger and Björkman, 1990). The operating efficiency of PSII photochemistry is determined by calculating ΔF/F_M_', also called Φ_PSII_ (Genty and Briantais, 1989; Murchie and Lawson, 2013). The possibility of measuring the incident photosynthetically active photon flux density (PPFD) with some PAM fluorimeters allows the calculation of another parameter, the estimated electron transport rate (ETR) (Flexas et al., 1999). The theoretical basis, assumptions regarding the parameters of PAM, and their calculations have been described in detail in the works of Genty and Briantais, 1989), Bilger and Björkman (1990); Maxwell (2000); Schreiber (2004); Baker (2008), and Murchie and Lawson (2013) (**Table 1**).

**Table 1:** Description of general and commonly used PAM chlorophyll fluorescence parameters.

Fluorescence parameters	Description	References
General parameters		
*F*_0_	initial fluorescence obtained in a dark adapted sample	Schreiber, 2004; Strasser et al., 2004
*F*_*M*_	maximum fluorescence after illumination of a dark adapted sample	Schreiber, 2004; Strasser et al., 2004
*F*_*V*_/*F*_*M*_ = (*F*_*M*_–*F*_0_)/*F*_*M*_	maximum quantum yield of PSII photochemistry	Schreiber, 2004; Strasser et al., 2004
Modulate fluorescence parameters		
*F*_0_'	minimal fluorescence yield measured shortly after darkening of an illuminated sample	Schreiber, 2004
*F*_*M*_'	maximum fluorescence after illumination of a light adapted sample	Schreiber, 2004
*F*_*S*_ = *F*_*t*_	steady-state value of fluorescence yield	Maxwell, 2000
*q*_*P*_ = (*F*_*M*_'–*F*_*S*_)/(*F*_*M*_'–*F*_0_')	photochemical quenching related to photochemical energy utilisation by charge separation at the reaction centres of PSII	Schreiber, 2004
*q*_*N *_= 1 – (*F*_*M*_' – *F*_0_')/(*F*_*M *_– *F*_0_)	non-photochemical quenching related to a rate of non radiative energy dissipation into heat	Schreiber, 2004
*NPQ* = (*F*_*M*_– *F*_*M*_')/*F*_*M*_'	non-photochemical quenching	Bilger and Björkman, 1990
*Φ*_*PSII*_ = *ΔF*/*F*_*M*_' = (*F*_*M*_' – *F*_*S*_)/*F*_*M*_'	operational efficiency of PSII photochemistry	Genty and Briantais, 1989
*ETR* = *Φ*_*PSII*_× *PPFD* × 0.5	electron transport rate	Flexas et al., 1999

The fast (or prompt) fluorescence analysis is based on the initial fluorescence signal after at least 20 minutes of dark adaptation of a leaf sample followed by a saturating pulse of actinic light (Strasser et al., 2004; Kalaji et al., 2014b). A fluorescence rise plotted on a logarithmic scale shows the so-called steps (L-, K-, J-, I-step) reflecting different phenomena occurring in and around PSII. This visualisation is often called the OJIP transient or the OJIP curve. The first part of the transient curve (O-J) expresses the photochemical events until the primary electron acceptor Q_A_ is reduced (Strasser et al., 2004; Bussotti et al., 2011a). The J-P section of OJIP transient (thermal phase) is related to electron transfer to end electron acceptors (Bussotti et al., 2011a). Prompt fluorescence analysis allows the assessment of the probability that a trapped exciton moves an electron into the electron transport chain beyond Q_A_ (ET_0_/TR_0_ = ψ_Eo_) and the probability that the moved electron reaches PSI acceptors (RE_0_/ET_0_ = δ_Ro_) (Strasser et al., 2004; Strasser et al., 2010). Additionally, specific energy fluxes expressed per active reaction centre (RC) and so-called ‘phenomenological energy fluxes per cross-section’ (CS) can be calculated, as absorption (ABS), trapping (TR_0_), thermal dissipation (DI_0_), electron transport rate beyond RC of photosystem II (ET_0_) and electron movement until end electron acceptors at the acceptor side of PSI (RE_0_). The parameter RC/CS reflects the total amount of active reaction centres per cross-section (Strasser et al., 2004). The analysis of OJIP parameters may be widened by analysis of additional steps on the OJIP curve, L-step, reflecting a decrease of energetic connectivity between PSII antennae, and K-step, which coincides with a limitation in the donor side of PSII (Strasser et al., 2004; Oukarroum et al., 2007). In many papers combined efficiency of electron transport up to end electron acceptors of PSI, δ_Ro_, and the efficiency of a movement of an electron into the electron transport chain beyond Q_A_, ψ_Eo_, appeared to be a good indicator of the stress response of plants (Bussotti et al., 2020). This combined parameter, denoted as ψ_REo_ or ΔV_IP_, shows the total efficiency of electron transport from PSII to PSI. Finally, two integrative parameters, so-called performance indices, were proposed by Strasser et al., 2004; Strasser et al., 2010, i.e. Performance Index on absorption basis (PI_ABS_) and total Performance Index (PI_total_). The calculations of all these parameters have been described in the papers noted above (**Table 2**).

**Table 2:** Description of commonly used prompt fluorescence (JIP-test) parameters.

Fluorescence parameters	Description	References
*F*_0_ = *ABS*/*CS*_0_	initial fluorescence obtained in a dark adapted sample	Strasser et al., 2004
*F*_*L*_ = *F*_150_	fluorescence at 150 μs after illumination of a dark adapted sample	Oukarroum et al., 2007
*F*_*K*_ = *F*_300_	fluorescence at 300 μs after illumination of a dark adapted sample	Strasser et al., 2004
*F*_*J*_ = *F*_2*ms*_	fluorescence at 2 ms after illumination of a dark adapted sample	Strasser et al., 2004
*F*_*I*_ = *F*_30*ms*_	fluorescence at 30 ms after illumination of a dark adapted sample	Strasser et al., 2004
*F*_*M*_ = *F*_*P*_	maximum fluorescence after illumination of a dark adapted sample	Strasser et al., 2004
*V*_*L*_ = (*F*_150_–*F*_0_)/(*F*_*M*_–*F*_0_)	relative variable fluorescence at 150 μs after illumination of a dark adapted sample	Oukarroum et al., 2007
*V*_*K*_ = (*F*_300_−*F*_0_)/(*F*_*M*_−*F*_0_)	relative variable fluorescence at 300 μs after illumination of a dark adapted sample	Strasser et al., 2004
*V*_*J*_ = (*F*_2*ms*_− *F*_0_)/(*F*_*M*_− *F*_0_)	relative variable fluorescence at 2 ms after illumination of a dark adapted sample	Strasser et al., 2004; Strasser et al., 2010
*V*_*I*_ = (*F*_30*ms*_− *F*_0_)/(*F*_*M*_− *F*_0_)	relative variable fluorescence at 30 mμs after illumination of a dark adapted sample	Strasser et al., 2004; Strasser et al., 2010
*V*_*K*_/*V*_*J*_	efficiency of electron flow from OEC to PSII reaction centres	Strasser et al., 2004; Strasser et al., 2010
*M*_0 _= 4 (*F*_300_− *F*_0_)/(*F*_*M*_− *F*_0_)	approximated initial slope of the fluorescence transient, expressing the rate of RCs’ closure	Strasser et al., 2004
*φ*_*Po *_= *TR*_0_/*ABS* = *F*_*V*_/*F*_*M*_= (*F*_*M*_−*F*_0_)/*F*_*M*_	maximum quantum yield of PSII photochemistry	Strasser et al., 2004
*ψ*_*o*_ = *ET*_0_/*TR*_0_= (*F*_*M*_– *F*_2*ms*_)/(*F*_*M*_– *F*_0_) = 1 – *V*_*J*_	probability that a trapped exciton moves an electron into the electron transport chain beyond Q_A_	Strasser et al., 2004; Strasser et al., 2010
*δ*_*Ro*_ = *RE*_0_/*ET*_0_= (*F*_*M*_– *F*_30*ms*_)/(*F*_*M*_– *F*_2*ms*_)	probability that an electron from the intersystem electron carriers is transferred to reduce end electron acceptors at the PSI acceptor side	Strasser et al., 2010
*ψ*_*REo*_ = *ΔV*_*IP*_= *ψ*_*Eo*_× *δ*_*Ro*_	total efficiency of electron transport from PSII to PSI	Strasser et al., 2010; Bussotti et al., 2020
*RC*/*ABS* = *γ*_*RC*_/(1 – *γ*_*RC*_) = *φ*_*Po*_(*V*_*J*_/*M*_0_)	Q_A_ reducing RCs per PSII antenna chlorophyll	Strasser et al., 2004
*RC*/*CS*_0 _= *φ*_*Po*_(*V*_*J*_/*M*_0_) (*ABS*/*CS*_0_)	density of active RCs (Q_A_ reducing RCs) per cross section at point 0	Strasser et al., 2004
*PI*_*ABS *_= *RC*/*ABS* × *φ*_*Po*_/(1 – *φ*_*Po*_) × *ψ*_*Eo*_/(1 – *ψ*_*Eo*_)	performance index (potential) for energy conservation from photons absorbed by PSII to the reduction of intersystem electron acceptors	Strasser et al., 2004
*PI*_*total*_ = *RC*/*ABS* × *φ*_*Po*_/(1 – *φ*_*Po*_) × *ψ*_*Eo*_/(1 – *ψ*_*Eo*_) × *δ*_*Ro*_/(1 – *δ*_*Ro*_)	performance index (potential) for energy conservation from photons absorbed by PSII to the reduction of PSI end electron acceptors	Strasser et al., 2010

In this paper, we review the research conducted to date on woody plants using ChF methods to monitor their response to different types of environmental stress. We have compiled the results of two of the most commonly used techniques performed at the foliar scale: PAM and prompt fluorescence, in particular, the JIP-test. The first part of the article describes experimental studies to identify stress factors affecting photosynthetic efficiency. Then, the role of photosynthetic efficiency screening in assessing tree vigour in natural and human-altered environments is analysed. Finally, we summarise the advantages and limitations of the ChF method in research on trees, shrubs, and woody vines.

## Chlorophyll *a* fluorescence measurements in laboratory and field experiments

ChF measurements, conducted to evaluate the effects of stress on the efficiency of the photosynthetic apparatus, are used to establish optimal conditions for crop production in the context of producing plant biomass, increasing yields, improving vigour, or selecting genotypes with greater resistance. Such research is widespread in annual crops such as wheat, rice, maize, and vegetables (Brestic and Zivcak, 2013; Kalaji et al., 2014a). However, there are also numerous papers describing experiments on woody plants (**Table 3**). The latter focused on crop production, the improvement of plant material for horticulture and urban greening, and applied studies in forest ecology.

**Table 3:** Chlorophyll *a* fluorescence measurements in stress detection in woody plants: species examined in the cited literature.

Stress factor	Reference	Examined species
Drought	Ögren, 1990; Percival and Fraser, 2002; Percival et al., 2006; Bacelar et al., 2007; Christen et al., 2007; Fini et al., 2009; Bussotti et al., 2010; Faraloni et al., 2011; Wang et al., 2012; Guha et al., 2013; Lee et al., 2016; Falqueto et al., 2017; Banks, 2018; Kalaji et al., 2018; Guadagno et al., 2021; Mihaljević et al., 2021; Fini et al., 2022	*Olea europaea; Acer platanoides*, *Acer pseudoplatanus*, *Acer campestre; Quercus petraea; Vitis vinifera; Olea europaea; Hevea brasiliensis; Tilia platyphyllos*, *Acer platanoides; Celtis australis*, *Fraxinus ornus; Pinus ponderosa*, *Populus tremuloides; Morus indica, Tilia cordata; Populus* ×*sibirica; Prunus avium; Salix* sp.; 9 *Fraxinus* species/cultivars; 30 woody species; *Vitis amurensis*
Light	Björkman and Powles, 1984; Brodribb and Hill, 1997; Hamerlynck, 2001; Gonҫalves et al., 2001; Lichtenthaler et al, 2004; Dias and Marenco, 2007; Cascio et al., 2010; Desotgiu et al., 2012a; Song and Li, 2016	*Nerium oleander; *Podocarpaceae family; *Fagus sylvatica; Minquartia guianensis; Ailanthus altissima; Platanus hybrida; Euonymus fortunei; Bombacopsis macrocalyx*, *Eugenia cumini*, *Iryanthera macrophylla*, *Senna reticulata*
UV-B radiation	Bavcon et al., 1996; Albert et al., 2005; Trošt Sedej and Rupar, 2013; Grifoni et al., 2016	*Salix arctica; Picea abies; Arbutus unedo*, *Vitis vinifera; Fagus sylvatica*, *Picea abies*
Heat	Percival, 2005; Duan et al., 2015; Esperon-Rodriguez et al., 2021	*Quercus ilex*, *Q. robur*, *Q. rubra; Malus domestica; Elaeocarpus reticulatus*, *Lophostemon confertus*, *Lagerstroemia indica*, *Liriodendron tulipifera*
Chilling, freezing	García-Plazaola et al., 1999; Jiang et al., 1999; Hakam et al., 2000; Percival and Fraser, 2001; Bussotti, 2004; Martínez-Ferri et al, 2004; Oliveira and Peñuelas, 2005; de Oliveira et al., 2009; Pflug and Brüggemann, 2012; Vitale et al., 2012; Míguez et al., 2017; Swoczyna et al., 2020	*Quercus ilex; Coffea arabica; Quercus ilex; Rosa rugosa*, *Rosa hybrida; Vitis labruscana; Juniperus phoenicea*, *Pinus halepensis*, *Q. ilex*, *Q. coccifera; *different subalpine species; *Cistus albidus*, *Quercus ilex*; 6 *Crataegus* species/cultivars; *Quercus ilex; *shrubs and herbaceous perennials, 23 species/cultivars; *Phillyrea angustifolia*
Chlorophyll defficiency	Torres Netto et al (2005); Percival et al., 2008; de Oliveira et al., 2009; Chen and Cheng, 2010; Swoczyna et al., 2010b; Castro et al., 2011	*Carica papaya; Malus domestica; Coffea arabica; Acer pseudoplatanus*, *Fagus sylvatica*, *Quercus robur; Acer campestre*, *Quercus rubra*, *Gleditsia triacanthos*, *Pyrus calleryana*, *Platanus ×hispanica* ‘Acerifolia’, *Ginkgo biloba*, *Tilia cordata*, *Tilia ×europaea*
Nitrogen defficiency	DaMatta et al., 2002; Percival et al., 2008; Nikiforou and Manetas, 2011; De Castro et al., 2014; Swoczyna et al., 2019	*Coffea canephora; Carica papaya; Pistacia lentiscus; Acer pseudoplatanus*, *Fagus sylvatica*, *Quercus robur; Actinidia arguta*
Phosphorus defficiency	Bosa et al., 2014	*Pyrus communis*
Salinity	Percival and Fraser, 2001; Percival et al., 2003; Percival, 2005; Naumann et al., 2008; Kalaji et al., 2018; Bashir et al., 2021	*Moringa oleifera; Tilia cordata; Myrica cerifera; Acer pseudoplatanus*, *Fagus sylvatica*, *Quercus robur; *30 *Acer* species/cultivars; *Quercus ilex*, *Q. robur*, *Q. rubra*
Ozone	Gerosa et al., 2003; Gravano et al., 2004; Bussotti et al., 2005; Bussotti et al., 2007a; Bussotti et al., 2007b; Gielen et al., 2007; Cascio et al., 2010; Desotgiu et al., 2012b; Gottardini et al., 2014; Pollastrini et al., 2014a	*Acer pseudoplatanus*, *Ailanthus altissima*, *Fagus sylvatica*, *Fraxinus excelsior*, *Viburnum lantana; Fagus sylvatica*, *Quercus robur*, *Populus nigra; Populus maximowiczii* × *P*. ×*berolinensis* (Oxford clone); *Viburnum lantana; Populus maximowiczii* × *P*. ×*berolinensis* (Oxford clone); *Viburnum lantana*, *Fraxinus excelsior*, *Populus nigra*, *Prunus avium*, *Quercus robur*
Gaseous air pollutants	Pukacki, 2000; Odasz-Albrigtsen et al, 2000; Alessio et al., 2002; Naidoo and Chirkoot, 2004; Matsushima et al., 2009; Fusaro et al., 2021 Wang et al., 2019	*Pinus pinea*, *Quercus ilex; Hibiscus* sp.; *Avicennia marina; Betula pubescens*, *Pinus sylvestris* and 5 shrub species; *Morus alba*
Heavy metals	Kitao et al., 1998; Pereira et al., 2000; Dezhban et al, 2015; Zhang et al., 2020; Hachani et al., 2021; Reyes et al., 2022	*Robinia pseudoacacia; Pinus halepensis; Betula ermanii*, *Alnus hirsuta; *4 *Citrus* species/cultivars; *Quercus ilex*, *Nerium oleander*, *Pittosporum tobira; Citrus grandis*, *Citrus sinensis*
Pests and pathogens	Percival and Fraser, 2002; Aldea et al., 2006; Christen et al., 2007; Percival, 2008; Cséfalvay et al., 2009; Muniz et al., 2014; Ugolini et al., 2014; Percival and Banks, 2015; Keča et al., 2018; Nowakowska et al., 2020	24 tree species; *Vitis vinifera; Fraxinus excelsior; Anacardium occidentale; Betula pendula; Aesculus hippocastanum*, *Quercus robur*, *Rosa rugosa; Malus* cv., *Castanea sativa*
Agrotechnical treatment	Bosa et al., 2014; Cirillo et al., 2021	*Pyrus communis; Olea europaea*
Urban paved surfaces	Philip and Azlin, 2005; Wang and Wang, 2010; Rahman et al., 2013	*Lagerstromia speciosa; Pyrus calleryana; Firmiana simplex*
Agrotechnical treatment	Bosa et al., 2014; Cirillo et al., 2021	*Pyrus communis; Olea europaea*

### Drought

Drought stress is the most commonly discussed problem in experiments using chlorophyll *a* fluorescence. Since water is the source of electrons used in the light-dependent photosynthetic process, the unimpeded availability of water may be critical for the successful conversion of light energy. Under moderate drought, the downregulation of the photosynthesis is mainly restricted by a decrease in stomatal conductance rather than the water-splitting reaction. Nevertheless, under severe drought the PSII efficiency may also be affected. Indeed, some experiments performed on detached leaves showed a correlation between the degree of dehydration and changes in the maximum quantum efficiency of PSII (Faraloni et al., 2011), whereas others did not (Ögren, 1990). These discrepancies may be due to different characteristics of taxa (species or varieties) and different biochemical mechanisms that ensure the balanced function of physiological processes. In the experiment described by Percival and Fraser (2002), nine of 30 ornamental taxa showed no significant changes in F_V_/F_M_ after 24-h dehydration.

From a practical point of view, information on whole-plant response is more useful in horticulture, plant breeding, evaluating suitability for urban environments, etc., because experiments conducted on whole plants reveal a plant’s overall strategy for coping with water deficiency. Indeed, the maximum quantum efficiency of PSII decreased during experimental drought stress in potted ornamental shrubs (Percival and Fraser (2002)), nine *Fraxinus* genotypes (Percival et al., 2006), six cultivars of *Olea europaea* L. (Faraloni et al., 2011), two cultivars of *Vitis amurensis* Rupr. (Wang et al., 2012), two clones of *Hevea brasiliensis* L. (Falqueto et al., 2017), *Tilia cordata* Mill. (Kalaji et al., 2018), two cultivars of *Prunus avium* L. (Mihaljević et al., 2021), 8-year-old *Olea europaea* trees in a commercial orchard (Bacelar et al., 2007) and two-year-old *Populus* ×*sibirica* seedlings planted in a reforestation area (Lee et al., 2016).

It should be noted, however, that in the laboratory, greenhouse and field experiments, the plants were generally treated with drought stress to the maximum water deficit in ambient light. Bukhov and Carpentier (2004) summarised several experiments and found that the maximum quantum efficiency was not strictly related to the water status of the plant and that moderate stress may not alter this parameter (Wang et al., 2012). Under low light, F_V_/F_M_ remained stable despite the reduced water potential of leaves, resulting in a decrease in stomatal conductance and CO_2_ assimilation rate in wheat (Lu and Zhang, 1998). These results suggest that drought stress exacerbates rather than triggers photoinhibition when there is an imbalance between light and water availability for photosynthetic performance (Bacelar et al., 2007). Moreover, the magnitude of changes in F_V_/F_M_ depends on the species or cultivar (Percival and Fraser, 2002; Wang et al., 2012). For example, the maximum quantum efficiency of PSII may not respond to drought in tolerant tree species (Fini et al., 2009; Swoczyna et al., 2010a; Fini et al., 2022).

In numerous experiments, the first symptom of drought stress is increased heat release of excess energy. In a 28-day experiment on *Tilia cordata*, Kalaji et al. (2018) found an increased dissipation rate after two weeks, which continued to rise in the following weeks. These results were also confirmed by Wang et al. (2012) and Lee et al. (2016). Similarly, drought treatment increased dissipation rates in different *Fraxinus* genotypes, but the effects were not the same across species and cultivars (Percival et al., 2006); these ChF parameters, including F_V_/F_M_, allowed the authors to rank 9 *Fraxinus* species and cultivars based on their drought resistance. The increase in F_0_ in response to drought was also found in *Vitis vinifera* L. (Christen et al., 2007) and *Hevea brasiliensis* (Falqueto et al., 2017). Fini et al. (2009) found significantly higher F_0_ in non-irrigated young *Tilia platyphyllos* L. and *Acer platanoides* L. trees during a dry July. Although the authors did not statistically compare the results between the lower and higher rainfall months, it clearly showed that F_0_ was significantly lower in *Tilia* species during a dry July (regardless of irrigation) than in June or August, when rainfall was more favourable. On the other hand, this finding can also be considered a synergistic effect of insufficient water availability and photoinhibition due to heat and high light conditions in July, the warmest month in northern Italy (Climate-data.org).

In addition to maximum quantum efficiency and dissipation parameters, there are other parameters that help detect drought stress, although F_V/_F_M_ does not clearly indicate the effects of stress (Bussotti et al., 2010). Faraloni et al. (2011) used the PAM instrument and found that ETR changed on the 14^th^ day of drought treatment and in the following days. The electron transport parameters downstream of PSII, ET_0_/RC, derived from OJIP analysis significantly decreased after two weeks, while ψ_Eo_ and φ_Eo_ decreased after three weeks of drought treatment in *Tilia cordata* (Kalaji et al., 2018). Guha et al. (2013) attributed the decrease in ET_0_/RC and ET_0_/CS_m_ to the maintenance of an intrinsic balance between electron transfer reactions and reductive carbon metabolism without severe damage to PSII in drought-resistant, five-month-old potted seedlings of *Morus indica* L. cultivar. On the other hand, Wang et al. (2012) found an increase in ET_0_/RC in drought-stressed *Vitis amurensis* and interpreted this as an acclimation response. Similarly, Lee et al. (2016) found unchanged ET_0_/RC and ET_0_/CS_0_ in *Populus* ×*sibirica* as a result of a compensatory mechanism.

Drought experiments revealed enhanced ABS/RC and TR_0_/RC (Wang et al., 2012; Falqueto et al., 2017; Mihaljević et al., 2021). This should be interpreted as the inactivation of PSII reaction centres, shown as reduced RC/CS_0_ by Lee et al. (2016) or RC/CS_m_ (Guha et al., 2013). However, in Christen et al. (2007) experiment ABS/CS_0_ and TR_0_/CS_0_, ABS/RC and TR_0_/RC values were significantly higher in drought-stressed *Vitis* plants, although RC/CS_0_ ratio was not significantly altered in relation to non-stressed plants.

Drought stress may also be detected by the appearance of L- and K-bands on OJIP transient, suggesting disturbances in energetic connectivity between PSII units and in the oxygen-evolving complex on the donor side of PSII, respectively. Falqueto et al. (2017) found positive L- and K-bands in one-year-old *Hevea brasiliensis* seedlings. However, they occurred only 36 days after drought treatment. Young *Tilia cordata* specimens showed the appearance of L- and K-bands on the 27^th^ day of drought treatment (Kalaji et al., 2018). Guha et al. (2013) found changes in L- and K-step on days 8 and 10 of drought in *Morus* saplings, while dissipation parameters increased since the 2^nd^ day of the experiment. On the other hand, Banks (2018) noticed the appearance of the K-band, but L-band was not evident. A clear K-step as a response to drought was noticed in one-year-old *Vitis amurensis* seedlings by Wang et al. (2012), but only in a drought-sensitive cultivar. Mihaljević et al. (2021) found the drought-induced appearance of L- and K-bands in *Prunus avium*, with a slight shift in the drought-tolerant cultivar and a strong response in the drought-sensitive one.

All the presented results indicate that water deficiency affects both the donor and acceptor sides of PSII, as well as the pool of reaction centres (Guadagno et al., 2021). In consequence, the performance indices PI_ABS_ and PI_total_, as integrative parameters, serve as good indicators of water deficit, as was shown by Guha et al. (2013); Falqueto et al. (2017) and Mihaljević et al. (2021). PI_ABS_ significantly decreased in drought-affected *Tilia cordata* on the 21st day of the experiment (Kalaji et al., 2018). Banks (2018) ascertained that PI_ABS_ responded to both drought and desiccation earlier than F_V_/F_M_.

### Light and UV-B radiation

Although light is the source of energy for plants, it is known that both too little and too much light can be a source of stress. Both leaves and chloroplasts are structurally adapted to the given light conditions (Lichtenthaler et al., 2004). For example, light-exposed and light-stressed leaves of trees have lower amounts of chlorophyll and smaller antennae. Lichtenthaler et al., 2004 found a higher maximum quantum efficiency in sun leaves than in shade leaves of *Fagus sylvatica* L., but this was not supported by Cascio et al. (2010) and Desotgiu et al. (2012a). The latter showed that trapping capacity was lower in light-exposed leaves of *Fagus sylvatica* seedlings than in shaded foliage, while electron transport efficiency to end-acceptors was higher beyond PSI. These properties allow balancing the energy flow between both photosystems and avoiding the formation of reactive oxygen species in case of electron excess. Changes in light conditions alter the performance of the photosynthetic apparatus. Under full sunlight at midday, the maximum quantum efficiency of PSII (F_V_/F_M_) decreases sharply (Dias and Marenco, 2007; Desotgiu et al., 2012a). The opposite trend was observed for the dissipation rate expressed by F_0_ (Dias and Marenco, 2007). Indeed, the ChF response in plants reflects their ecophysiological characteristics. In shade-grown and shade-tolerant plants, smaller values of PPFD may saturate non-photochemical quenching (qN), whereas, in species with high light requirements, a saturation of qN may not occur even at high maximum daily irradiances (Brodribb and Hill, 1997). A shade-tolerant mahogany (*Swietenia macrophylla* King) showed higher F_0_ values, especially in sun-exposed leaves, but lower F_M_ and F_V_ when exposed to strong light, in contrast to the sun-tolerant tonka bean (*Dipteryx odorata* (Aubl.) Willd.), which had similar F_0_ values in both sun-exposed and shaded seedlings (Gonҫalves et al., 2001). These results indicate that ecophysiological traits are an important factor to consider when interpreting fluorescence results. Light stress may not affect PSII alone. Björkman and Powles (1984) found that daylight combined with water stress resulted in increased photoinhibition in *Nerium oleander* L., whereas shaded leaves showed no changes in the primary photochemistry of PSII.

UV-B radiation regulates various processes in plants, but it may also have a negative impact on photosynthetic efficiency. In fact, Bavcon et al. (1996) found that UV-B radiation combined with low temperatures affected F_V_/F_M_ and net photosynthetic activity in *Picea abies* (L.) Karst. UVB may determine the breakdown of OEC and enhancement of the K-band (Grifoni et al., 2016). As stated by Day et al. (1992), the resistance to UV-B radiation is higher in coniferous trees than in deciduous species. The seasonal changes in susceptibility to UV-B radiation were noted by Albert et al. (2005) in *Salix arctica* Pall. In late season limitation of the natural dose of UV-B was not visible, while in July, natural radiation resulted in diminished maximum quantum efficiency of PSII (F_V_/F_M_), the estimated number of reaction centres (RC/CS_m_), rate of electron transport beyond PSII (ET_0_/TR_0_, ET/CS_m_) and, in consequence, PI_ABS_, compared to specimens with limited access to UV-B. Likewise, Trošt Sedej and Rupar (2013) found a seasonal influence of enhanced UV-B radiation on F_V_/F_M_ in seedlings of *Fagus sylvatica* and *Picea abies*.

### Extreme temperatures

Heat stress initially increases heat dissipation, as reflected by an increase in F_0_, and decreases the maximum quantum efficiency of PSII (Percival, 2005; Duan et al., 2015). OJIP analysis by Duan et al. (2015) demonstrated the complexity of the effects of heat stress by showing perturbations in OEC, reaction centre pool, and electron transport to the end of electron acceptors PSI. However, young leaves studied at the beginning of the growing season are more susceptible to heat stress. There are also differences between genotypes, e.g., leaves of *Quercus ilex* L. (an evergreen species) are more resistant to heat, while those of *Q. robur* L. and *Q. rubra* L. (deciduous) are more susceptible (Percival, 2005). The susceptibility of PSII to heat stress, expressed by an elevated F_0_ value, was used by Esperon-Rodriguez et al. (2021) to determine critical temperatures for studying heat tolerance in urban trees.

Both cold and frost stress adversely affect physiological processes in plants. Chilling decreases the quantum efficiency of PSII (Hakam et al., 2000; de Oliveira et al., 2009), but the effect depends on the species characteristics (Oliveira and Peñuelas, 2005). Percival and Fraser, (2001) studied the effects of freezing and salt in six *Crataegus* genotypes. As a result of freezing stress, decreases in F_V_/F_M_ and PI_P_ were associated with increases in heat release (F_0_). Chlorophyll *a* fluorescence was also used to evaluate the woody tissue viability of Concord grapevine (*Vitis labruscana* Bailey) after controlled frost stress (Jiang et al., 1999). The ratio F_V_/F_M_ correlated well with freezing temperatures and leaf tissue damage. Evergreen Mediterranean plants exposed to winter stress often show reduced maximum quantum efficiency and quantum yield of PSII electron transport (Φ_PSII_) (Vitale et al., 2012). In contrast, Swoczyna et al. (2020) found that neither F_V_/F_M_ nor PI_ABS_, but parameters related to PSII reaction centres, showed significant correlations with winter survival of woody plants and perennials cultivated in a vertical garden on the wall of an urban building. However, according to Pflug and Brüggemann (2012), dissipation and absorption rates were negatively correlated with minimum temperatures in evergreen *Quercus ilex*, whereas the correlation of F_V_/F_M_ and ET_0_/TR_0_ with T_min_ was positive. This suggests that frost not only slows down the processes but also affects the structures of the photosynthetic apparatus. The sensitivity of PSII to winter stress in *Quercus ilex* was confirmed by Bussotti (2004) in forest stands when morning photoinhibition was observed as a reduced number of active reaction centres (RC/CS_0_), F_V_/F_M_ and performance index PI_ABS_. Photoinhibition caused by low temperatures and concomitant high solar radiation is more pronounced in broadleaf evergreen species (angiosperms) than in conifers or semi-deciduous species in Mediterranean habitats (García-Plazaola et al., 1999; Martínez-Ferri et al., 2004). However, in response to winter stress, woody plants show a more conservative strategy than herbaceous species to survive the damaging period (Míguez et al., 2017), involving different phenomorphological adaptations and protective biochemical mechanisms.

### Chlorophyll content

Numerous studies in plants have shown that photosynthetic efficiency is usually associated with adequate levels of photosynthetic pigments (de Oliveira et al., 2009; Swoczyna et al., 2010b). Torres Netto et al (2005) found that a reduction in chlorophyll content in leaves resulted in a decreased fluorescence emission, as reflected by a change in the values of some parameters: F_M_ and F_V_/F_M_, with only chlorophyll-rich leaves showing optimal values for F_V_/F_M_. Similar observations were made by Percival et al. (2008); regardless of the species studied, relative chlorophyll content of SPAD-502 below 25 resulted in a decrease in F_V_/F_M_. Chen and Cheng (2010) studied 7-year-old apple trees in an orchard with foliar chlorosis. Compared to normal leaves, chlorotic leaves exhibited increased deactivation of oxygen-evolving complexes (OEC), minimal fluorescence (F_0_), dissipated energy, and relative variable fluorescence at L, K, J, and I bands. Simultaneously, maximum fluorescence (F_M_) and quantum yields, i.e. maximum quantum yield for primary photochemistry (F_V_/F_M_ = TR_0_/ABS), quantum yield for electron transport (ET_0_/ABS) and quantum yield for the reduction of end acceptors of photosystem I (PSI) (φ_Ro_ and RE_0_/ABS) were decreased. Likewise, the maximum amplitude of the IP phase, the density of active reaction centres of PSII (RC/CS_0_) and performance indices (PI_total,_ PI_ABS_) were diminished. This means that photoinhibition occurred at both the donor (i.e., the OEC) and the acceptor sides of PSII in chlorotic leaves. However, the acceptor side was damaged more severely than the donor side, which possibly was the consequence of the over-reduction of PSII due to the slowdown of the Calvin cycle. Castro et al. (2011) showed a clear positive relationship between the results of optically determined chlorophyll content and RC/CS_0,_ while in the case of ABS/RC and TR_0_/RC, the relationship was negative.

### Nutrient availability

The insufficiently available element whose deficiency is most frequently detected by the ChF method is nitrogen (N). An important constituent of amino acids, nitrogen plays an essential role in protein synthesis and in numerous biochemical processes in the form of enzymes, including light and dark reactions in chloroplasts (Lawlor, 2002). Nitrogen is also a component of chlorophyll, so N deficiency is clearly indicated by decreases in chlorophyll content (De Castro et al., 2014) and negatively affects net CO_2_ assimilation rates (DaMatta et al., 2002; De Castro et al., 2014). DaMatta et al. (2002) studied the effect of abundant and limited nitrogen fertilisation on *Coffea canephora* Pierre plants. N limitation resulted in a slight decrease in F_V_/F_M_, a more significant decrease in photochemical quenching and operational quantum efficiency (qP and Φ_PSII_, respectively), and an increase in non-photochemical quenching (NPQ) in well-watered plants. However, there was no significant difference in NPQ and other parameters due to N availability in water-deficient plants. Percival et al. (2008) showed that low N content in leaves, which is strongly linked to chlorophyll content, leads to a decrease in F_V_/F_M_. An optimum of F_V_/F_M_ was found at leaf N contents of at least 1%, 1.5%, and 2% in *Acer pseudoplatanus*, *Fagus sylvatica*, and *Quercus robur*, respectively (Percival et al., 2008)

Nutritional factors were assessed by Nikiforou and Manetas (2011) on *Pistacia lentiscus* L. in field conditions. These authors concluded that nitrogen deficiency affected the parameters related to the I-P phase. This relationship was visible independently of the season, while parameters related to the PSII activity (i.e. quantum yields for photon trapping and electron flow along PSII and the efficiency of a trapped exciton to move an electron from the first plastoquoinone electron acceptor of PSII to intermediate carriers) were limited by low nitrogen only during the winter period.

On the other hand, in a field study by Swoczyna et al. (2019) on *Actinidia arguta* (Sieb. & Zucc.) Planch. ex Miq. grown on a commercial plantation, the results suggested lower dependence of the performance of end electron acceptors around PSI upon N content while the effect of the ‘climate-conditions × N-treatment’ combination on the PSII performance was higher. During the more favourable season the differences in N-treatment were well pronounced in V_K_/V_J_, RC/ABS, F_V_/F_M_, ψ_Eo_, PI_ABS_, and PI_total_. The most sensitive parameter to N nutrition was the density of active RCs per cross-section (RC/CS_0_) as it allowed the distinction effects of N-treatment independently of the season. The similar patterns of both RC/CS_0_ and RC/ABS differences suggested these parameters to be good indicators for N deficiency.

The strong dependence of photosynthetic efficiency on N availability is not shared in the case of other nutrients. According to Bosa et al. (2014), potassium fertilisation did not show significant effects on the light energy conversion process in pear trees grown in an experimental orchard.

### Salt stress

Salt stress affects plants in a similar manner to drought stress, causing osmotic limitations in water uptake, tissue desiccation, and hyperionic and hyperosmotic stress in cells. If salinity persists, additional stress leads to toxic effects on photosynthesis and other important metabolic processes (Chaves et al., 2009). The response of photosynthetic efficiency to salt stress has been studied at both the leaf and whole plant levels.

Percival and Fraser, 2001 used ChF to examine foliar salt tolerance in detached leaves in 6 *Crataegus* genotypes. Initial fluorescence F_0_ increased in three taxa in response to increasing salinity, while F_V_/F_M_ and PI_P_ decreased in 5 genotypes. The authors explained the PI_P_ parameter in the next publication (Percival et al., 2003) as a calculation of RC/ABS × φ_Po_/(1 – φ_Po_) × ψ_o_/(1 – ψ_o_), thus it may be identified as PI_ABS_. The combined freezing × salt stress had a serious negative effect in all six genotypes. Differences in PI_P_ response to salt between detached leaves of 30 *Acer* genotypes facilitated the ranking of *Acer* genotypes according to their salt tolerance (Percival et al., 2003). In that examination F_0_ and F_V_/F_M_ did not give such clear results.

On the other hand, young potted and field-grown *Quercus* trees revealed clear changes of F_V_/F_M_ and F_0_ as a response to sodium chloride solution applied as a spray to the foliage (Percival, 2005). Salt stress-induced gradual decline in maximum quantum efficiency (F_V_/F_M_) and the increase in F_0_, the most pronounced reaction to stress treatment in young trees, occurred in the 3-4th week after treatment. The time necessary to recover from salt damage was the 12^th^ (*Q. ilex*, *Q. rubra*) or 14^th^ week (*Q. robur*). These findings demonstrate that, at the level of the whole plant, the response to salt stress is delayed due to (1) slow and gradual accumulation of salt and (2) mobilisation of metabolic processes towards defence and/or acclimation to stress (Chaves et al., 2009). The tendency to maintain the high maximum quantum efficiency of PSII during stress conditions was found in numerous research. Naumann et al. (2008) noticed that F_V_/F_M_ was diminished significantly in salt-flooded potted seedlings of *Myrica cerifera* L. (a shrub species sensitive to salt stress) only after noticeable damage to leaves. However, other PAM parameters were better indicators: stress was effectively detected through the decrease of ΔF/F_M_' and increase of Φ_NPQ_ prior to visible signs. Thus, the calculation of parameters other than F_V_/F_M_ gives more information and allows the detection of stress at an earlier stage of its occurrence. In the experiment by Bashir et al. (2021) on 4-week-old seedlings of *Moringa oleifera* Lam. two levels of NaCl stress showed alterations in ChF in comparison to control. In light-adapted samples, parameters of PAM fluorometry Y(II) decreased while NPQ increased. Quantum yield of non-photochemical fluorescence quenching by non-dissipation energy, Y(NO), increased by 25% and 80% at a lower and higher level of salt stress, respectively, indicating that in highly stressed seedlings, both photochemical energy conversion and protective regulatory mechanisms were inefficient in protection against photodamage. Additionally, analysed OJIP parameters, PI_ABS_, φ_Po_ (=F_V_/F_M_) and ψ_Eo_, decreased in stressed plants, while ABS/RC had already increased with the lower stress level. In the experiment of Kalaji et al. (2018) on *Tilia cordata* potted saplings, salt stress significantly reduced maximum fluorescence, F_M_, on the 14^th^ day of the experiment, causing a decrease of F_V_/F_M_ (= φ_Po_) in the next days. On the 21^st^ day of the experiment φ_Do_ and ET_0_/RC were changed significantly. Finally (on the 28^th^ day), most of both donor (DI_0_/RC, φ_Do_ and K-step) and acceptor PSII side parameters (ET_0_/RC, φ_Eo_, ψ_o_) were significantly changed. In general, in that experiment, the results of salt stress were similar to drought stress. However, the principal component analysis revealed a separate arrangement of the salt and drought stress cases. The cases of drought stress were more or less directly along PC1 and its determinants, whereas the plotting of salt stress appeared more disorderly, with the pattern changing with increasing salt pressure. This suggests that salt stress more strongly affects the various structures and/or physiological processes around PSII.

### Ozone, air pollution, soil contamination

The effects of the tropospheric ozone (O_3_) as a pollutant on chlorophyll fluorescence traits were one of the main questions raised in experimental and field studies, as reviewed by Bussotti et al, (2007b); Bussotti et al, (2011a). As a general result, F_V_/F_M_ was demonstrated to be quite insensitive, at least in the first phases of ozone treatment, whereas the most sensitive parameters were those related to the I-P phase and the concentration of reaction centres per cross-section (RC/CS_0_). NPQ was the main parameter connected with ozone impacts in modulated fluorescence. Experimental studies were carried out on young trees (seedling and potted plants) in open-top chambers facilities, both with enriched and ambient ozone pollution levels, to screen the relative sensitivity of different species such as *Viburnum lantana* L., *Fraxinus excelsior* L., *Populus nigra* L., *Prunus avium* and *Quercus robur* (Gravano et al., 2004). In these experiments, it was probed that the intensity of the responses was related to leaf structure, with higher sensitivity in species with high SLA and in sunny exposed leaves (Gerosa et al., 2003; Bussotti et al., 2007a; Cascio et al., 2010). The sensitive poplar clone *Populus maximowiczii* Henry × *P*. ×*berolinensis* Dippel (Oxford clone) was adopted as a model plant to study the mechanisms of ozone damage with the application of chlorophyll fluorescence techniques (Desotgiu et al., 2012b; Pollastrini et al., 2014a). In field studies, the main subject was the responses connected to visible foliar symptoms. Bussotti et al. (2005) found that species-specific behaviours were connected to the de-excitation mechanisms. Such mechanisms were related to the irreversible damage of PSII in *Ailanthus altissima* (Mill.)Swingle and a more effective quenching capacity (as a process of compensative photosynthesis) in *Fraxinus excelsior* and *Acer pseudoplatanus*. In the experimental field site of Kranzberger (Germany), where tall *Fagus sylvatica* trees were subjected to artificial ozone treatment, Gielen et al. (2007) found only a limited decrease in the quantum yield efficiency. Gottardini et al. (2014) observed the pattern of ChlF on *Viburnum lantana* shrubs with different levels of ozone symptoms. Symptomatic plants showed significantly lower values of the maximal fluorescence (F_M_), the maximum quantum yield of primary photochemistry (F_V_/F_M_), J phase and Performance Index Total (PI_TOT_ = PI_total_), according to the most used abbreviation showed in the **Table 2** and significantly higher values of minimal fluorescence (F_0_) throughout the growing season, respect to non-symptomatic plants.

Other gaseous air pollutants may have different effects on photosynthetic efficiency. Sulphur dioxide (SO_2_) had negative effects on the maximum quantum efficiency of PSII due to its toxic effect on leaf tissue (Pukacki, 2000; Matsushima et al., 2009). On the other hand, low atmospheric NO_2_ pollution may serve as an additional N source for plants and consequently increase photosynthetic efficiency (Wang et al., 2019). Unfortunately, studies on the effects of gaseous pollutants on photosynthetic efficiency investigated with ChF are sparse.

In several studies, ChF was used to assess the impact of heavy metal contamination on PSII performance. Kitao et al. (1998) examined Mn toxicity in two-year-old potted seedlings of four deciduous broad-leaved tree species differing in successional traits using PAM fluorescence. The authors confirmed differences between early-successional species (*Betula ermanii* Cham. and *Alnus hirsuta* Turcz.) having a higher tolerance to excessive accumulations of Mn in leaves than two other mid- and late-successional species. The toxicity of aluminium salts decreased maximum quantum efficiency in citrus genotypes (Pereira et al., 2000; Zhang et al., 2020). The efficiency of electron transport beyond PSII reaction centres was diminished in *Citrus grandis* L. only, while *Citrus sinensis* L. did not respond to Al treatment (Zhang et al., 2020). Likewise, Dezhban et al. (2015) ascertained the negative impact of cadmium and Pb chloride on F_V_/F_M_ and increased F_0_ values in one-year-old *Robinia pseudoacacia* L. seedlings. On the other hand, the ChF method allowed to confirm the beneficial effect of ectomycorrhizal fungi on recovery from contamination stress (Pb, ZN and Cd) in *Pinus halepensis* Mill. (Hachani et al., 2021). The research on soil pollution influence was conducted mostly on crop plants. However, it would be interesting to gain more knowledge on the impact of heavy metal contamination on trees with regard to the accumulation of contaminants.

### Pests and pathogens

Arthropod herbivory and pathogen infections alter plant physiological processes in different ways depending on which parts of the plant are damaged. Damage to vascular tissue and leaf vein reduces water supply to photosynthesizing cells and alters nutrient and osmotic transport, cell content feeding reduces photosynthesis, and defoliation damage can disrupt the water balance in remaining tissues, and release of biocidal compounds against attackers can alter photosynthetic and homeostatic mechanisms, while some pathogens and pests can also produce toxins that directly or indirectly affect photosynthetic metabolism or produce compounds that act as plant growth regulators (Nabity et al., 2009; Rolfe and Scholes, 2010). Welter (1989, cited by Nabity et al., 2009) found that over 50% of all plant-insect interactions resulted in a loss of photosynthetic capacity. On the other hand, in some cases, local injury contributes to increased CO_2_ assimilation in remaining tissues or organs (Nabity et al., 2009).

Because the ChF method is non-invasive, it allows, in combination with other methods, e.g., gas exchange, thermal imaging, UV imaging, the tracking of plant-pathogen interactions throughout the life cycle of a pathogen and indirect effects of pathogens and herbivorous arthropods on the photosynthesis of a host plant (Aldea et al., 2006). The contribution of chlorophyll fluorescence imaging techniques to understanding metabolic changes in plants due to biotic hazards has been discussed in reviews by Nabity et al. (2009), arthropod herbivory, and Rolfe and Scholes (2010), pathogen infections, as well as in the recent work by Pérez-Bueno et al. (2019). Biotic injury on leaves is generally scattered across the leaf surface, and likewise vascular constraints, leads to specifically localised changes in leaf chemistry, which is why the fluorescence imaging technique is often used to map changes in infected leaves or plants in laboratory research. This technique allows the identification of sites for pathogen or herbivore activity but also enables the analysis of conventional parameters: F_0_, F_M_, F_V_/F_M_, Φ_PSII_, q_N_, q_P_, NPQ (Pérez-Bueno et al., 2019). Cséfalvay et al. (2009) used a kinetic imaging fluorometer to detect the effect of artificial inoculation with *Plasmopara viticola* (Berk. & M.A. Curtis) Berl. & De Toni (the causal agent of downy mildew) on *Vitis vinifera* leaves. The distribution of changed F_V_/F_M_ and Φ_PSII_ across the leaf lamina was associated with the presence of the developing mycelium three days before the occurrence of visible symptoms and five days before the release of spores. The reduction of maximum quantum efficiency of PSII (reflecting the injury of PSII complexes) was restricted to the leaf area that later yielded sporulation, while the area with significantly lower Φ_PSII_ (often correlated with the yield of CO_2_ fixation) was larger. Three types of interactions between host and pathogen are possible: biotophic, deriving nutrients from living cells and maintaining their viability, necrotrophic, destroying host cells and digesting its tissues, and hemi-biotrophic, initially feeding on nutrients from living cells and then feeding as necrotrophs (Scholes and Rolfe, 2009). In many studies on biotrophs and hemi-biotrophs changed photosynthetic efficiency was detected in asymptomatic tissues as an announcement of the disease development, with Φ_PSII_, q_P_ and NPQ being more sensitive pre-symptomatic signals of infection than F_V_/F_M_. The timing of changes in the above-mentioned parameters in necrothophs was more variable, and NPQ appeared to be more valuable for pre-symptomatic signalling (Pérez-Bueno et al., 2019). Host leaves may not show any changes in photosynthetic efficiency except in infected sites and surrounding areas, destruction of vascular tissues in woody plants may reduce water availability for leaf cells, as well as nutrients and assimilates supplied to all plant tissues. Muniz et al. (2014) investigated the early symptoms of *Lasiodiplodia theobromae* (Pat.) Griffon & Maubl. (an endophyte colonising stem tissues) in two-month-old *Anacardium occidentale* L. seedlings inoculated with pathogen mycelium. The infection significantly changed the maximum and operational quantum efficiency of PSII (F_V_/F_M_ and Φ_PSII_), both photochemical and non-photochemical quenching (q_P_ and NPQ), prior to visible symptoms, which may be attributed to the limitations of water supply.

The fast-fluorescence method provides several sensitive parameters which may be useful in the early detection of infection. Early research was done by Percival and Fraser (2002), who studied changes in PSII performance in woody species (ornamental rose, oak and horse chestnut) infected by powdery mildew agents (*Sphaerotheca pannosa* (Wallr.) Lev. var. *rosae* Wor., *Phyllactinia* sp., *Uncinula necator* (Schwein.) Burrill), biotrophic fungi. Photosynthetic CO_2_ fixation tended to be reduced prior to the visible signs of infection, while changes in F_0_ and F_V_/F_M_ were visible when the mycelium had covered more than 25% of the leaf blade. However, the performance index calculated on the basis of the OJIP curve showed a decrease when the first symptoms of infection were visible (less than 10% of the leaf blade covered with the mycelium). Pathogens developing in the vascular system also influenced photosynthetic efficiency in asymptomatic leaves in 16-year-old *Vitis vinifera* L. plants grown in a vineyard (Christen et al., 2007) before confirming the symptoms of white rot and necrosis on the basis of wood decay. An early stage of the esca disease was signalled by a significant increase in dissipation, expressed by DI_0_/RC, DI_0_/CS_0_ and φ_Do_, and a decrease in φ_Po_, ψ_Eo_, and PI_ABS_. Infection diminished a pool of active reaction centres (RC/CS_0_) and electron transport rates (ET_0_/RC and ET_0_/CS_0_) in infected plants but not significantly. Likewise, increased dissipation DI_0_/CS_0_ and decreased φ_Po_, ψ_Eo_, PI_ABS_ and PI_total_ were shown by Keča et al. (2018) in the research on *Fraxinus excelsior* L. seedlings inoculated with *Hymenoscyphus fraxineus* Baral et al. and *Phytophtora* spp. Nowakowska et al. (2020) investigated the interactions between two hazardous pathogens *Phytophtora cactorum* (Lebert & Cohn) J. Schröt., *Armillaria gallica* Marxm. & Romagn. and *Betula pendula* Roth. seedlings, the authors noticed that the pathogen infection increased thermal dissipation of energy absorbed by PSII (via shifted DI_0_/RC and DI_0_/CS_0_) but also downregulated electron transport beyond primary acceptors (as is shown by a decrease of ψ_Eo_=ET_0_/TR_0_) and diminished the number of active reaction centres.

In some papers, ChF is used to evaluate the effect of chemical control in infected plants. Percival (2008) assessed the effectiveness of paclobutrasol as a fungicid against *Venturia inaeqalis* (Cooke) G. Wint.), and *Guignardia aesculi* (Peck) VB Stewart) on *Malus* cv. Crown Gold and *Aesculus hippocastanum* L., respectively. The application of paclobutrasol had a positive effect on the visually evaluated leaf health status and the photosynthetic efficiency expressed by performance index (PI) values calculated from chlorophyll *a* fluorescence measurements. Percival and Banks (2015) applied the ChF technique to investigate the effect of preventative and curative treatment with potassium or silicon phosphite on the health condition of *Aesculus hippocastanum* saplings inoculated with *Pseudomonas syringae* pv. *aesculi*. That experiment gave the perception that preventative treatment had a greater protecting effect than the application three weeks after the inoculation.

### Rootstock effect and agrotechnical treatments

Finally, the ChF method is a sensitive and rapid tool for screening the effects of evolving agrotechnical practices. The type of rootstock affected the photosynthetic efficiency of grafted pear trees. The higher F_V_/F_M_ and PI_ABS_ values indicated that the rootstock type provided better photosynthetic productivity of the grafted cultivar, which was confirmed by higher chlorophyll content and net photosynthetic rate (Bosa et al., 2016). Cirillo et al. (2021) studied the effect of biostimulants, kaolin (administered as Manisol by Manica S.p.a, Rovereto, Italy) and di-1-p-mentene (administered as Vapor Gard^®^ by Biogard^®^, Bergamo, Italy), on two-year-old potted olive seedlings during a hot summer. F_V_/F_M_ proved to be a sufficient parameter for evaluating the usefulness of these anti-transpiration products.

## Chlorophyll *a* fluorescence measurements in the natural environment and urban landscape

**Figure 1:**
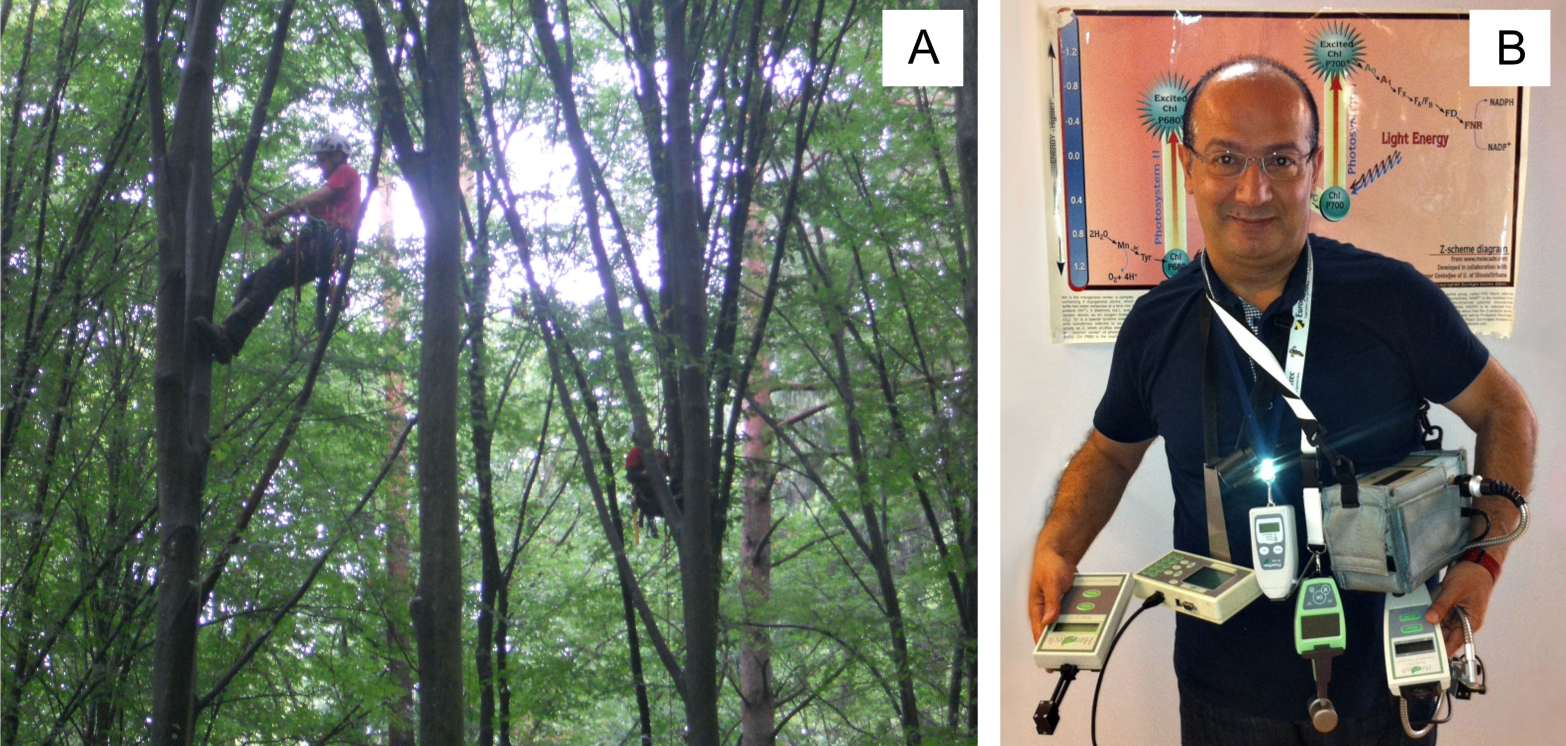
The sampling for chlorophyll fluorescence measurement at the Bialowieza forest (2013) for the FunDiv EUROPE project, photo: F. Bussotti **(A)**. The various available portable fluorometers to be used in forestry and other scientific disciplines, photo: H.M. Kalaji **(B)**.

With the development of portable fluorimeters, ChF technology has opened new opportunities for *in situ* research (**Figure 1**). Extensive experience in experimental research has provided the basis for investigations and interpretation of results at experimental sites where environmental conditions were not well defined and where multiple stress effects are expected. Such studies are important because they provide the opportunity to learn how plants actually function in a natural environment. They also allow monitoring of the condition of plants in a man-made environment, such as a city, where human attention is not usually focused on plant well-being. The results of these studies are particularly important for trees, on which carbon sequestration, habitat maintenance, local climate regulation and, in cities, human well-being depend.

### Assessing a tree

Sampling and measuring chlorophyll fluorescence parameters on the leaves of mature trees in forests or urban parks poses several problems. Leaves can be difficult to reach, and measurements at canopy height are not readily possible unless trees are scaffolded; therefore, it is preferable to work on detached leaves.

Sampling techniques for leaves from tall trees include the use of loppers, tree climbers, and shooting, depending on tree height, crown structure, and local operational constraints (Bussotti and Pollastrini, 2015b). The number of leaves to be sampled depends on the variability of the assessed parameters between and within trees (Gottardini et al., 2014). Leaves should be randomly sampled within the crown (to represent the entire tree) or concentrated in a particular stratum, e.g., from the top only (to reduce the source of variability). Significant differences between sun and shade leaves (top and bottom of the canopy, respectively) were observed in forest trees for the parameters F_V_/F_M_ and I-P phase (Pollastrini et al., 2017), combining lower values of F_V_/F_M_ with higher values of IP phase in sun leaves.

Chlorophyll fluorescence parameters show a typical diurnal pattern (Zhang and Gao, 2000). The high intensity of solar radiation leads to photoinhibition of the photosynthetic apparatus with depression of F_V_/F_M_ at midday (Epron et al., 1992; Kalaji et al, 2017) Under the same light conditions, there may be an increase in electron transport beyond photosystem I (PSI) (Pollastrini et al., 2017). Therefore, leaves should be sampled and measured at similar times of day, or dynamic and chronic photoinhibition should be eliminated (or at least reduced) by long dark adaptation (at least 4-5 h) so that leaves collected at different times of day are comparable (Pollastrini et al., 2016a).

Percival and Fraser (2002) have shown that there is no difference between the results of intact and detached leaves when they are protected from dehydration stress. Therefore, users often collect leaves from plants and perform measurements under laboratory conditions (Bussotti and Pollastrini, 2015b) or even in the field but in a shaded area (Swoczyna et al., 2019). The possibility of taking samples for later measurements facilitates the task when samples from tall trees are difficult to obtain.

Variability in chlorophyll fluorescence parameters is an important consideration when planning a field survey. In a pan-European survey, Pollastrini et al. (2016a), F_V_/F_M_ proved to be very stable within a tree (coefficient of variation, CV = 1.42 within the crown, in 16 sampled leaves) and between trees (coefficient of variation, CV = 1.46 in 6 sampled trees), whereas composite indices (i.e., performance indices in the JIP test) show large variability (PI_ABS_: CV = 29.81 with six trees sampled). In general, ratios and normalised parameters (fluxes and yields) are less variable than the original ChF signals, such as F_0_ and F_M_. This is an important aspect of the comparability of results from different fluorimeters (Bussotti et al., 2011b). ChF parameters correlate with each other and can be grouped into clusters in terms of the information they provide. Bussotti et al. (2020) suggest that in large-scale surveys, overall photochemical efficiency can be represented by two independent parameters, F_V_/F_M_ and I-P phase, which is representative of photosystem II (PSII) and PSI efficiency, respectively.

The ChF signal is determined by the age of a leaf and its phenological stage. Young and senescent leaves have different ChF properties than mature, fully developed leaves due to incomplete assembly of the photosynthetic machinery and degradation of chlorophyll and photosystems (Jiang et al., 2006a; Jiang et al., 2006b; Lepeduš et al., 2010; Holland et al., 2014; Duan et al., 2015; Sitko et al., 2019). Lepeduš et al. (2010) observed that changes in F_V_/F_M_ in ageing leaves were less pronounced than changes in PSII capacity for O_2_ evolution determined using a gas-phase oxygen electrode system. Holland et al. (2014) found that the appearance of the K-band indicated disturbances in the oxygen-evolving complex but at the later stage of ageing. It has also been noted that during the growing season, the strength of the ChF signal may decrease (Swoczyna et al., 2020; Suchocka et al., 2021), reflected in decreased F_0_ and F_M_ in the late season, and is not caused by leaf physiology, but rather by morphological changes, i.e., thickening of the cuticle, etc. In evergreen conifers and deciduous trees, differences between the different age classes are to be expected.

### Assessing a forest

Chlorophyll fluorescence analysis is widely used in forest research (Epron et al., 1992; Aldea et al., 2006; dos Santos and Ferreira, 2020) but rarely directly on tall trees in forest ecosystems and for operational purposes (Ball et al, 1995), although it provides important insights into photosynthesis and plant physiology (Mohammed et al., 1995). Forests are complex ecosystems with a stratified structure of woody and herbaceous plant species that include mature trees, shrubs, herbs, regeneration, and epiphytes, each with a different size and life span.

Among the publications dealing with the analysis of active chlorophyll fluorescence of forest trees, important scientific findings come from the studies conducted within the FP7 project “FunDivEUROPE - The functional significance of forest tree diversity in Europe” (Baeten et al., 2013). In this project, the ChF characteristics of trees in six European forests, from the Mediterranean to the boreal, were assessed. The data presented by Pollastrini et al. (2016a) show that different tree species growing in the same site have specific chlorophyll fluorescence signatures (with differences between conifers and deciduous trees and between early- and late-growing species), while ChF characteristics change in the same species growing in different sites. Moreover, photosynthetic performance assessed by ChF was higher in central European forests than in southern (Mediterranean) and northern (Boreal) borders. In mixed stands, the main factor that changed ChF parameters was inter-tree competition: dominant trees were more affected by photoinhibition of leaves in the upper part of the canopy, with a reduction of F_V_/F_M_, than leaves in the lower part of the canopy (Bussotti and Pollastrini, 2015a).

The ChF analysis on forest trees was applied to investigate the health condition of forests (Odasz-Albrigtsen et al, 2000). Special attention was paid to the relationships between defoliation and ChF parameters. Partial defoliation allows the penetration of light into the crown, then allowing better exploitation of sunlight energy, but, at the same time, induces photoinhibition processes of the PSII (Gottardini et al., 2016; Gottardini et al., 2020). A rise in the electron transport rate beyond the PSI compensates for the reduction of F_V_/F_M_ with species-specific patterns, as shown by Pollastrini et al, (2014b); Pollastrini et al, 2016c; Pollastrini et al., 2017). *Castanea sativa* Mill. trees defoliated by the insect *Dryocosmus kuriphilus* (Asian chestnut gall wasp) demonstrated the reduction of the IP phase in the infected leaves (Ugolini et al., 2014). Based also on these results, ChF analysis has been proposed as a tool to integrate the current activities concerning the assessment of the conditions of forests in the monitoring networks (Bussotti and Pollastrini, 2017) within the ICP Forests programme (http://icp-forests.net).

### Assessing urban forests and trees

Both ChF techniques, PAM and prompt ChF, have been used for stress detection in urban environments and human-altered habitats, such as degraded areas. Such sites are characterised by variable edaphic and microclimatic conditions and are usually quite different from natural habitats. ChF analysis makes it possible to detect stress in urban trees before visible signs appear (Swoczyna et al., 2010b; Ugolini et al., 2012; Reyes et al., 2022) or to indicate which specimens are particularly affected by road stress (Hermans et al., 2003). In this way, ChF can be a tool for identifying specimens that need more intensive care. ChF also provides arguments for better design of public spaces that provide suitable growing conditions for trees. Highly compacted soils (Philip and Azlin, 2005), artificially created pits for tree plantings (Rahman et al., 2013), and impermeable soil surfaces (Wang and Wang, 2010) negatively affect photosynthetic efficiency. Urban trees and other plants are expected to improve environmental conditions for human well-being, assimilate CO_2_, and provide shade and aesthetic values. Air and soil pollution and dust deposition on leaves reduces photosynthetic efficiency (Alessio et al., 2002; Naidoo and Chirkoot, 2004; Popek et al., 2018; Fusaro et al., 2021; Reyes et al., 2022). Other environmental factors, e.g., shading or excessive light in open areas such as plazas and parking lots, can also be assessed using the ChF method (Hamerlynck, 2001; Song and Li, 2016), keeping in mind that different types of stress can have a synergistic or additive effect on photosynthetic efficiency (Fusaro et al., 2021). Finally, the ChF technique allows the selection of species tolerant to urban environments (Swoczyna et al., 2010b; Swoczyna et al, 2015; Reyes et al., 2022). Assessment of photosynthetic efficiency helps explain the successful adaptation of selected alien species that can easily adapt to unfavourable conditions; this knowledge is particularly important in the case of invasive alien species (Mlinarić et al., 2021).

In numerous papers, only F_0_, F_M_, and F_V_/F_M_ were used as indicators of stress. This sometimes resulted in weak or no changes in ChF in stressed trees, in contrast to, for example, stomatal conductance (Martinez-Trinidad et al., 2010; Benson et al., 2019). Analysis of parameters describing the donor side and electron transport on the acceptor side of PSII, in particular OJIP analysis, provides more sensitive indicators of environmental stress in the case of moderate changes (Bussotti et al., 2010; Ugolini et al., 2012). It should also be considered that, as a living organism in a given habitat, a tree tends to maintain an adequate state of photosynthetic structures to provide sufficient nutrition to all parts of the organism. Thus, damage in one part of a tree can lead to the upregulation of photosynthetic output in another part through what is known as compensatory photosynthesis (Martinez-Trinidad et al., 2010; Suchocka et al., 2021).

## Conclusions: Advantages and limitations

The most important advantage of using chlorophyll fluorescence is that it provides a tool for objective evaluation of the photosynthetic efficiency of trees. The collection of a large amount of comparable data in forest tree communities is, therefore, crucial for the early diagnosis of changes in plant vigour, as it allows many samples to be examined *in situ* over a short time. Among ChF techniques, the JIP assay is a powerful tool for *in vivo* analysis of plant stress (Strasser et al., 2000; 2004) that has been widely used in plant physiology and ecology research for many decades and has been applied in forest ecology research (Gottardini et al., 2014; Pollastrini et al., 2016a; Pollastrini et al., 2016b; Pollastrini et al., 2016c; Pollastrini et al, 2017).

Chlorophyll fluorescence has been successfully used in applied research to evaluate the effects of stressors on tree seedlings and small plants and to screen genotypes adapted to specific environmental conditions (Kuhlgert et al., 2016; Çiçek et al., 2020; Dimitrova et al., 2020). ChlF analysis has been used by Bantis et al. (2020); Bantis et al. (2021); Bashir et al., (2021) and Pollastrini et al. (2020) to select tree species for reforestation under climate change conditions by analysing the results of a system of community gardens across Europe. ChF is also applied in nurseries to determine young tree vigour and potential seedling performance (Perks et al., 2001; L’Hirondelle et al., 2007), stand quality (Binder et al., 1996), and winter hardiness (Fisker et al., 1995). The main limitation is the time required for dark adaptation of the leaves, but when working with detached leaves, this problem can be overcome.

Due to technical and operational constraints, active fluorescence techniques (i.e., the use of artificially generated actinic light) are not widely applied in tall tree research, whereas there is increasing interest in the application of passive (sun-induced) fluorescence through remote sensing techniques (from satellite to UAV, Rossini et al., 2006; Yang et al., 2017; Mohammed et al., 2019). Remote sensing surveys evaluate the optical properties of foliage to assess parameters such as leaf area index, chlorophyll content, and photosynthetic efficiency (Serbin et al., 2012). In the Sentinel 3/FLEX programme, passive chlorophyll fluorescence (ChlF) emitted by vegetation is assessed to evaluate the state of vegetation across Europe (Mohammed et al., 2019). Photosystem functionality is considered an indicator of photosynthetic efficiency (Baker and Oxborough, 2004). In remote sensing studies, ChlF parameters are associated with the net primary production of both terrestrial and aquatic ecosystems (Norton et al., 2019). However, we believe that active fluorescence can play an important role in answering specific tree-level questions (passive fluorescence provides surface-level data) and validating remote observations.

## Author contributions

Review of the literature: TS, FB, MP; manuscript revision: FB, MP, HK; final preparation: TS, HK, JM. Percentage contribution of the Authors to the manuscript preparation is as follows: TS = 70%, MP = 20%, FB = 5%, JM = 3% and HK = 2%. All authors contributed to the article and approved the submitted version.
